# Evaluation of Blood Pressure and Heart Rate Measurements from Smartwatch HUAWEI WATCH D2 Data: A Preliminary Report

**DOI:** 10.3390/healthcare14213566

**Published:** 2026-10-22

**Authors:** Octavio A. Lecona, Vania J. Martínez-Garcés, Bruno Estañol, Antonio Barajas-Martinez, Paola V. Olguín-Rodríguez, Ruben Fossion, Ana Leonor Rivera

**Affiliations:** 1 Instituto de Ciencias Nucleares, Universidad Nacional Autónoma de México, Mexico City 04510, Mexico; tavolecona@ciencias.unam.mx (O.A.L.); vania.mtzgarces@gmail.com (V.J.M.-G.); fossion@nucleares.unam.mx (R.F.); 2 Plan de Estudios Combinados en Medicina (PECEM-MD/PhD), Facultad de Medicina, Universidad Nacional Autónoma de México, Mexico City 04510, Mexico; 3 Departamento de Neurofisiología Clínica, Instituto Nacional de Ciencias Médicas y Nutrición Salvador Zubirán, Mexico City 14080, Mexico; bruno.estanolv@gmail.com; 4 Escuela de Medicina y Ciencias de la Salud, Tecnológico de Monterrey, Monterrey 64849, Mexico; antonio.barajas@tec.mx; 5 Centro de Investigación en Ciencias, Universidad Autónoma del Estado de Morelos, Cuernavaca 62209, Mexico; paola.olguin@uaem.mx

**Keywords:** blood pressure monitoring, smartwatch validation, wearable devices

## Abstract

**Highlights:**

**What are the main findings?**

- In a Mexican cohort, the HUAWEI WATCH D2 showed close agreement with values from standard devices for heart rate and acceptable agreement for blood pressure in the seated position.
- Device performance depended on posture, wrist placement, and measurement conditions, with lower blood pressure agreement in the upright position.

**What are the implications of the main findings?**

- Wearable devices may enable scalable cardiovascular assessment through repeated spot measurements in real-world settings.
- Standardized and robust measurement protocols may improve the reliability of wearable blood pressure assessment and cardiovascular risk evaluation.

**Abstract:**

**Background/Objectives:** Wearable devices offer a scalable approach to repeated cardiovascular assessment through ambulatory spot measurements and have demonstrated adequate performance in measuring heart rate (HR). However, their reliability for blood pressure (BP) assessment remains uncertain. This study aimed to evaluate BP and HR measurements obtained with the HUAWEI WATCH D2 smartwatch by comparison with standard reference devices under controlled postural conditions. **Methods:** A total of 30 participants (15 women and 15 men; age range, 21–88 years) were enrolled. One participant could not undergo smartwatch measurement because of wrist-size limitations, and measurement acquisition failed in another participant. Consequently, paired measurements obtained in the seated and upright positions were analyzed for 28 participants. Smartwatch-derived systolic BP (SBP), diastolic BP (DBP), and HR were compared with a digital sphygmomanometer, an analog sphygmomanometer, and a digital pulse oximeter. Agreement was assessed using percentage error and Bland–Altman analysis, complemented by linear regression to evaluate proportional bias. **Results:** In the seated position, the smartwatch showed agreement with percentage errors below 4% for SBP and DBP, with concordance coefficients indicating good agreement. In the upright position, accuracy decreased, with percentage errors up to 14%, increased variability, wider limits of agreement, and the presence of systematic and proportional discrepancies. Measurement performance was influenced by device placement, and isolated failures highlighted sensitivity to anatomical and technical factors. **Conclusions:** Smartwatch-based BP monitoring is feasible under controlled conditions but remains dependent on posture and measurement conditions. These findings support the use of wearable devices for scheduled spot-check cardiovascular assessment under controlled conditions and underscore the need for standardized measurement protocols before their implementation in ambulatory or long-term monitoring applications.

## 1. Introduction

High blood pressure is the most prevalent modifiable risk factor for cardiovascular disease and is associated with coronary artery disease, heart failure, stroke, chronic kidney disease, and all-cause mortality [1,2,3]. Accordingly, hypertension represents a major global public health challenge, affecting approximately 1.4 billion adults aged 30–79 years worldwide, while only a minority of patients achieve effective (BP) control [4,5]. In this context, accurate and accessible BP assessment is essential for cardiovascular prevention, diagnosis, and follow-up [1,3,4]. Repeated monitoring of physiological variables has been increasingly proposed as a tool for assessing health status within preventive medicine. In cardiovascular physiology, variables such as BP and (HR) provide insight into the functional state of the system and its underlying regulatory dynamics. Traditionally, BP assessment relies on conventional cuff-based sphygmomanometers, which provide punctual readings under specific measurement conditions, whereas invasive intra-arterial catheters allow continuous recording but are not suitable for routine or out-of-office use. These limitations have motivated the development of cuffless and wearable approaches for BP monitoring, although important challenges related to accuracy, calibration, and clinical validation remain [6].

BP is not a static variable, but the result of dynamic interactions among multiple regulatory mechanisms. Postural transitions, such as the change from seated or supine to upright position, induce a redistribution of blood volume driven by gravity, leading to transient BP changes that are compensated by coordinated cardiovascular responses, including baroreflex activation and local myogenic vasoconstriction [7]. This intrinsic variability reflects the adaptive nature of cardiovascular homeostasis and imposes constraints on the reliability of measurement systems, particularly under non-static conditions [8]. Therefore, accurate BP assessment requires standardized procedures that account for patient preparation, body position, cuff size, measurement environment, device validation, and the number of readings obtained [3]. However, these recommendations are not always consistently implemented in routine clinical practice; it was recently reported that primary care physicians measured BP in only one third of their patients, and only 21.3% performed three measurements per visit [9]. Together, these physiological and methodological considerations highlight the need for accessible, validated tools capable of supporting repeated BP assessment under controlled and standardized conditions.

Traditional approaches for BP measurement based on isolated readings may fail to capture its temporal dynamics [10]. Out-of-office BP monitoring, including ambulatory BP monitoring (ABPM) and home BP monitoring (HBPM), provides information beyond office BP by increasing the number of measurements and improving the estimation of an individual’s usual BP levels [3]. ABPM allows repeated measurements over a 24 h period, whereas HBPM offers a more practical and accessible approach for longitudinal BP assessment in clinical practice [3]. Devices such as Portapres^®^ can also be used for continuous non-invasive BP monitoring under controlled conditions [11]. In this context, out-of-office or ambulatory use refers to non-invasive BP assessment outside highly specialized clinical settings, such as home-based or scheduled measurements, rather than unrestricted beat-to-beat monitoring during daily life. Smartwatches stand out due to their accessibility and their ability to acquire physiological data non-invasively. Wearable devices may expand monitoring capabilities by enabling repeated or scheduled measurements outside highly specialized clinical settings [1,12]. However, their use in clinical or preventive contexts requires careful performance evaluation to determine accuracy, agreement with reference devices, and potential systematic differences under defined measurement conditions [13,14,15].

Smartwatches have demonstrated high accuracy in specific tasks, such as predicting physical performance, showing strong agreement between predicted and observed outcomes [12], particularly in running [16]. In clinical settings, wearable devices have also facilitated remote monitoring strategies, enabled the detection of cardiac events, and supported post-intervention follow-up in cardiovascular patients [17]. Additionally, their capacity to record physiological signals, particularly heart rhythm and HR, has positioned them as useful tools for the detection of arrhythmias and other cardiovascular alterations [18]. However, performance cannot be assumed to be equivalent across all physiological variables. The clinical implementation of wearable devices remains limited by the need for rigorous validation; although agreement with reference instruments may be acceptable for certain measurements, performance varies as a function of the target variable, device technology, physiological condition, measurement protocol, and population studied [19]. Furthermore, large-scale observational data indicate that, although wearable devices are widely adopted, their effective use and long-term adherence are heterogeneous, highlighting challenges in their integration into routine health monitoring [20]. Therefore, the utility of these devices depends on the accuracy and reliability of their measurements compared with clinical reference methods, particularly for BP assessment.

Among available devices, the HUAWEI WATCH D2 incorporates an inflatable cuff mechanism, representing an intermediate approach between conventional sphygmomanometers and wearable technologies. Previous studies comparing this device with validated clinical instruments reported moderate agreement and identified limitations in specific populations, particularly in normotensive individuals [21]. More recently, studies evaluating the HUAWEI WATCH D2 reported acceptable accuracy according to international validation standards in resting, ambulatory, and nocturnal BP measurements, as well as potential advantages related to comfort and reduced sleep disruption compared with conventional ABPM [22,23]. However, independent evaluations under real 24 h ambulatory conditions also reported insufficient concordance for BP measurements, despite reliable HR performance [24]. These contrasting findings underscore the need for further validation under controlled conditions and in different populations, particularly considering the physiological variability associated with posture, wrist position, and measurement setting.

In this context, the aim of the present study was to evaluate cardiovascular measurements obtained using a wearable device, the HUAWEI WATCH D2, by comparing them with reference measurements acquired using conventional clinical devices under controlled conditions.

## 2. Materials and Methods

An exploratory study was conducted at the Physics of Life Laboratory in the Institute of Nuclear Sciences of the Universidad Nacional Autónoma de México, to evaluate the concordance between blood pressure and heart rate measurements obtained with a wrist-worn consumer smartwatch HUAWEI WATCH D2 and a clinical reference instrument.

The cohort comprised 30 participants (15 women and 15 men) aged 21–88 years. Non-invasive measurements were performed in accordance with the Declaration of Helsinki after all participants provided written informed consent to participate in the study and for the scientific use of the data collected.

Systolic blood pressure (SBP), diastolic blood pressure (DBP), and heart rate (HR) were recorded with a HUAWEI WATCH D2 smartwatch (SW; Huawei Technologies Co., Ltd.^®^, Shenzhen, China) worn on the left wrist. Measurements were obtained in the seated position and after 2 min in the upright position. The data are available in the Supplementary Material.

The digital oscillometric sphygmomanometer (DS; UA-751, A&D Company, Limited, Tokyo, Japan) was defined a priori as the primary reference device because it was positioned on the same arm as the smartwatch, thereby minimizing physiological variability related to inter-arm differences. The analog aneroid Hergom^®^ sphygmomanometer (AS; B1-# CheckATek, Hergom Medical, Mexico City, Mexico) positioned on the contralateral arm, was included as a secondary comparator to assess agreement across measurement modalities and to provide an independent reference placed on the same arm (left), and an analog aneroid (AS) Hergom^®^ sphygmomanometer placed on the right arm. Device resolution for BP was 1 mmHg for DS and 2 mmHg for AS; pulse-oximeter HR resolution was 1 bpm, while smartwatch reported 3 mmHg for BP and 1 bpm for HR.

Oxygen saturation (SpO_2_) and HR were recorded using a digital fingertip pulse oximeter. SpO_2_ was collected as a descriptive physiological measure and was not included as an outcome in the BP agreement analysis; the corresponding values are available in the Supplementary Material. Pulse-oximeter HR was used as a clinical comparator for an exploratory device-specific assessment of smartwatch HR. Because no electrocardiographic reference was used, the study was not designed as a formal validation of smartwatch-derived HR.

Measurements were obtained sequentially rather than simultaneously. For each participant and postural condition, oxygen saturation and heart rate were first recorded using the pulse oximeter. Blood pressure and heart rate were then measured with the HUAWEI WATCH D2 smartwatch, followed by the digital oscillometric sphygmomanometer (primary reference device) and finally by the analog aneroid sphygmomanometer (secondary comparator). Simultaneous inflation of cuff-based devices was intentionally avoided to prevent mechanical interference and potential alterations of local hemodynamics.

### Statistical Analysis

Bland–Altman analysis was used to evaluate the agreement between the smartwatch and the gold reference devices. For each comparison, the mean of paired measurements and their differences were computed to estimate the bias and the limits of agreement (mean difference ± 1.96 standard deviations). Throughout, the sign convention is Δ = reference-smartwatch, so that a negative bias denotes the smartwatch reading as being higher than the reference. Linear regression of the differences as a function of the means was also performed to assess the presence of proportional bias. This approach allows the identification of systematic deviations and the evaluation of measurement accuracy under the studied conditions. We complemented this analysis by the reference-axis regression [25,26] that regresses the paired differences on the reference measurement rather than on the coupled pair mean, thereby avoiding the mathematical coupling that can produce spurious or masked proportional-bias slopes in the conventional formulation. Agreement was additionally summarized with Lin’s concordance correlation coefficient (CCC) and its 95% confidence interval obtained by non-parametric bootstrap (R = 2000 resamples).

For the same-arm smartwatch-versus-digital comparisons, Deming regression (λ = 1, with Jackknife confidence intervals) and Passing–Bablok regression (median of pairwise slopes, with bootstrap confidence intervals, R = 2000) were also computed to examine systematic proportionality between devices. Measurement-system uncertainty from device resolution was treated as a Type B contribution under a uniform-distribution assumption, the standard uncertainty contributed by each device is δ/√12 (where δ is the full-scale resolution), and the combined resolution-limited uncertainty of a paired difference is their quadrature sum, yielding a 95% floor of ±0.80 mmHg for SW-versus-DS comparisons and ±1.27 mmHg for comparisons involving the analog device.

## 3. Results

Measurements could not be obtained for one participant (ID F8301) due to technical constraints. The participant’s small wrist circumference prevented proper smartwatch placement, and no appropriately sized cuff was available for the digital sphygmomanometer.

For another participant (ID M8801), smartwatch measurements were unsuccessful in both seated and upright positions, even after switching wrists and smartwatches. The participant had a normal body mass index and no apparent health conditions; the cause of this issue remains undetermined.

Accurate smartwatch placement proved critical for reliable measurements, requiring appropriate strap adjustment to avoid both excessive tightness and looseness.

All participants exhibited blood pressure and heart rate values within normal physiological ranges in both seated and upright positions. Measurements across all subjects are shown in Figure 1.

We calculated percentual measurement errors as the value detected by smartwatch-reference detected by medical device; the results are summarized as box plots in Figure 2, while medians and interquartile ranges for key comparisons are reported in Table 1. The largest discrepancies were observed between the smartwatch and sphygmomanometers under the upright condition, particularly for SBP and DBP; HR comparisons showed substantially smaller discrepancies across both positions. The cross-arm agreement between the two cuff references was excellent in the seated position (DS − AS bias +0.1 mmHg; CCC 0.918 [0.88, 0.94]), indicating negligible inter-arm differences in this cohort.

In Figure 3, DS vs. SW (SBP) comparison top-left panel (a) shows that the bias is negative (≈−4 mmHg), indicating that the smartwatch tends to report higher systolic values than the digital device. The limits of agreement are wide (approximately −20 to +12 mmHg), reflecting substantial dispersion, but Lin’s concordance correlation coefficient is 0.81 [0.70, 0.87], indicating strong seated concordance. The regression line shows a slight negative slope, suggesting a minimal decrease in the difference as the mean pressure increases; however, the trend appears weak, indicating no relevant proportional bias. The reference-axis regression confirms the absence of proportional bias on either axis. Deming and Passing–Bablok regressions both yield slopes whose 95% confidence intervals include the unity—Deming 1.04 (0.80, 1.28); Passing–Bablok 0.89 (0.67, 1.12)—consistent with proportional agreement between the devices and a constant offset.

For the DS vs. SW (DBP) comparison, shown in the top-right panel of Figure 3, the bias is close to zero or slightly negative (≈−1 mmHg), indicating minimal systematic difference between the digital device and the smartwatch for diastolic pressure. The limits of agreement are narrower than in the digital comparison (approximately −13 to +11 mmHg), suggesting improved agreement (approximately 30% narrower than for SBP) and CCC = 0.80 (0.64, 0.88). The regression line shows a slight positive slope, indicating a weak increase in the difference with increasing pressure, though without a clear proportional pattern.

In the AS vs. SW (SBP) comparison, shown at the bottom-left panel of Figure 3, the bias remains negative (≈−4 to −5 mmHg), again indicating higher values from the smartwatch relative to the analog device. The dispersion is larger than in the digital-vs-smartwatch comparison (panel a), with wider limits of agreement (approximately −22 to +13 mmHg) and CCC = 0.77 (0.66, 0.85). The regression slope is slightly negative, suggesting a small tendency for the difference to decrease at higher systolic values, although the effect remains weak.

Finally, in the AS vs. SW (DBP) comparison, shown at the bottom-right panel of Figure 3, the bias is slightly positive (≈+1 mmHg) but not statistically significant, indicating that the smartwatch tends to report slightly lower diastolic values than the analog device. The limits of agreement are moderate (approximately −12 to +14 mmHg) and CCC = 0.77 (0.61, 0.86). The regression slope is positive, suggesting a small increase in the difference at higher pressure values, although, as in the other panels, the effect appears limited.

Figure 4 shows the Bland–Altman plots in the upright position for four device comparisons. From left to right, the panels correspond to the following: digital sphygmomanometer vs. smartwatch for systolic blood pressure (DS vs. SW, SBP), digital vs. smartwatch for diastolic pressure (DS vs. SW, DBP), analog sphygmomanometer vs. smartwatch for systolic pressure (AS vs. SW, SBP), and analog vs. smartwatch for diastolic pressure (AS vs. SW, DBP). In all panels, black points represent paired measurements, the red horizontal line indicates the bias, blue dashed lines the 95% limits of agreement (LoA), and the purple line the linear regression used to assess proportional bias. Agreement deteriorates substantially under the upright condition: across all four panels, the limits of agreement widen, and concordance coefficients fall, with the LoA width ratio between upright and seated reaching 2.03 for the DS vs. SW (SBP) comparison and the CCC dropping from 0.81 to 0.45 for the same pair.

In the DS vs. SW (SBP) comparison, top-left panel (Figure 4), the bias is markedly negative (≈−10 mmHg), indicating that the smartwatch systematically reports higher systolic values than the digital device in the upright position. The limits of agreement are wide (approximately −40 to +20 mmHg, 2-times the width of the seated comparison), indicating high dispersion, with CCC = 0.45 (0.12, 0.65). The regression line shows a positive slope, suggesting that the difference increases with higher pressure levels, indicating the presence of proportional bias. The reference-axis regression provides quantitative confirmation of proportional bias. Both Deming and Passing–Bablok regressions depart from proportional agreement under upright conditions, with the Passing–Bablok slope 0.52 (0.23, 0.78) excluding unity and its intercept +68.9 mmHg (37.6, 107.6) excluding zero.

For DS vs. SW, top-right panel (Figure 4), the bias remains negative (≈−8 mmHg), again indicating higher values from the smartwatch. The dispersion is substantial, with limits of agreement around −30 to +15 mmHg and CCC = 0.28 (0.04, 0.54), indicating markedly weaker agreement than in the seated position. The regression slope is negative, suggesting that the difference becomes more negative at higher diastolic values, indicating a weak but consistent proportional effect. However, the reference-axis regression suggests a pattern consistent with the mathematical coupling of the conventional regression rather than a genuine dependence on the underlying diastolic pressure. Both Deming and Passing–Bablok regressions are affected by the high variance of the upright DBP: Deming confidence intervals are very wide, slope 95% CI [−0.54, 5.15], and Passing–Bablok estimates with a slope of 0.68 (0.07, 1.10), broadly consistent with proportional agreement but with substantial uncertainty.

In the AS vs. SW (SBP) comparison, bottom-left panel (Figure 4), the bias is also negative (≈−12 mmHg), showing that the smartwatch reports higher systolic values than the analog device. The limits of agreement are wide (approximately −40 to +15 mmHg), similar to the digital comparison, and CCC = 0.42 (0.17, 0.59). The regression line is nearly flat, indicating no clear proportional bias despite the systematic offset. The reference-axis regression, in contrast, detects a significant proportional bias, present but masked by the mathematical coupling of the conventional formulation.

Finally, in the AS vs. SW (DBP) comparison bottom-right panel (Figure 4), the bias is negative (≈−10 mmHg), indicating higher diastolic values from the smartwatch relative to the analog device. The limits of agreement remain broad (approximately −30 to +12 mmHg) and CCC = 0.30 (0.09, 0.48). The regression line shows a negative slope, indicating that the difference becomes more pronounced at higher pressure levels, consistent with proportional bias. Nonetheless, the reference-axis regression does not support proportional bias, a pattern parallel to panel (b).

## 4. Discussion

The −4.4 mmHg DS vs. SW offset is of the magnitude expected for the wrist-to-brachial pulse-wave amplification gradient, which typically produces a 3–6 mmHg increases in peak systolic pressure at distal sites [26], and therefore reflects a physiological pressure gradient rather than a device error.

Measurements depended on proper device placement, highlighting the importance of standardized positioning and strap adjustment. Additionally, difficulty in obtaining measurements in specific cases reveals the limitations associated with anatomical factors and device characteristics.

Future work will focus on continuous blood pressure monitoring over a 3-day period using the smartwatch, with the aim of identifying early warning signals of cardiovascular dysregulation. This approach is supported by the observation that smartwatch measurements exhibit errors below 10%.

The smartwatch showed acceptable agreement with the primary digital reference sphygmomanometer under controlled seated conditions. Comparisons with the secondary analog sphygmomanometer yielded similar trends, supporting the robustness of the observed findings, with percentage errors generally below 10% in seated position measurements. However, larger discrepancies were observed under the upright condition, particularly for systolic and diastolic blood pressure, indicating sensitivity to postural changes and measurement conditions. The results suggest that the smartwatch may represent a useful tool for scheduled blood pressure spot-check measurements outside traditional clinical settings. However, the present study does not evaluate unrestricted ambulatory monitoring, and further studies are required to determine performance during daily activities and long-term use; however, their use requires careful consideration of measurement conditions and device limitations.

## 5. Conclusions

The HUAWEI WATCH D2 SW demonstrated acceptable agreement with reference sphygmomanometers for blood pressure measurement under controlled seated conditions, with percentage errors below 4% for both systolic and diastolic values and concordance coefficients indicating strong agreement. However, measurement accuracy degraded substantially in the upright position, with increased variability, wider limits of agreement, and the emergence of systematic and proportional biases.

The results indicate that smartwatch-derived blood pressure measurements are highly dependent on postural conditions and device placement. The observed systematic offsets are consistent with known physiological pressure gradients between measurement sites, while the increased dispersion under upright conditions reflects the influence of dynamic cardiovascular regulation.

These findings establish that smartwatch-based blood pressure monitoring is feasible under controlled conditions but remains context-dependent. Accurate implementation in clinical or preventive settings requires standardized protocols for repeated spot measurements and careful consideration of the physiological state.

## Figures and Tables

**Figure 1:**
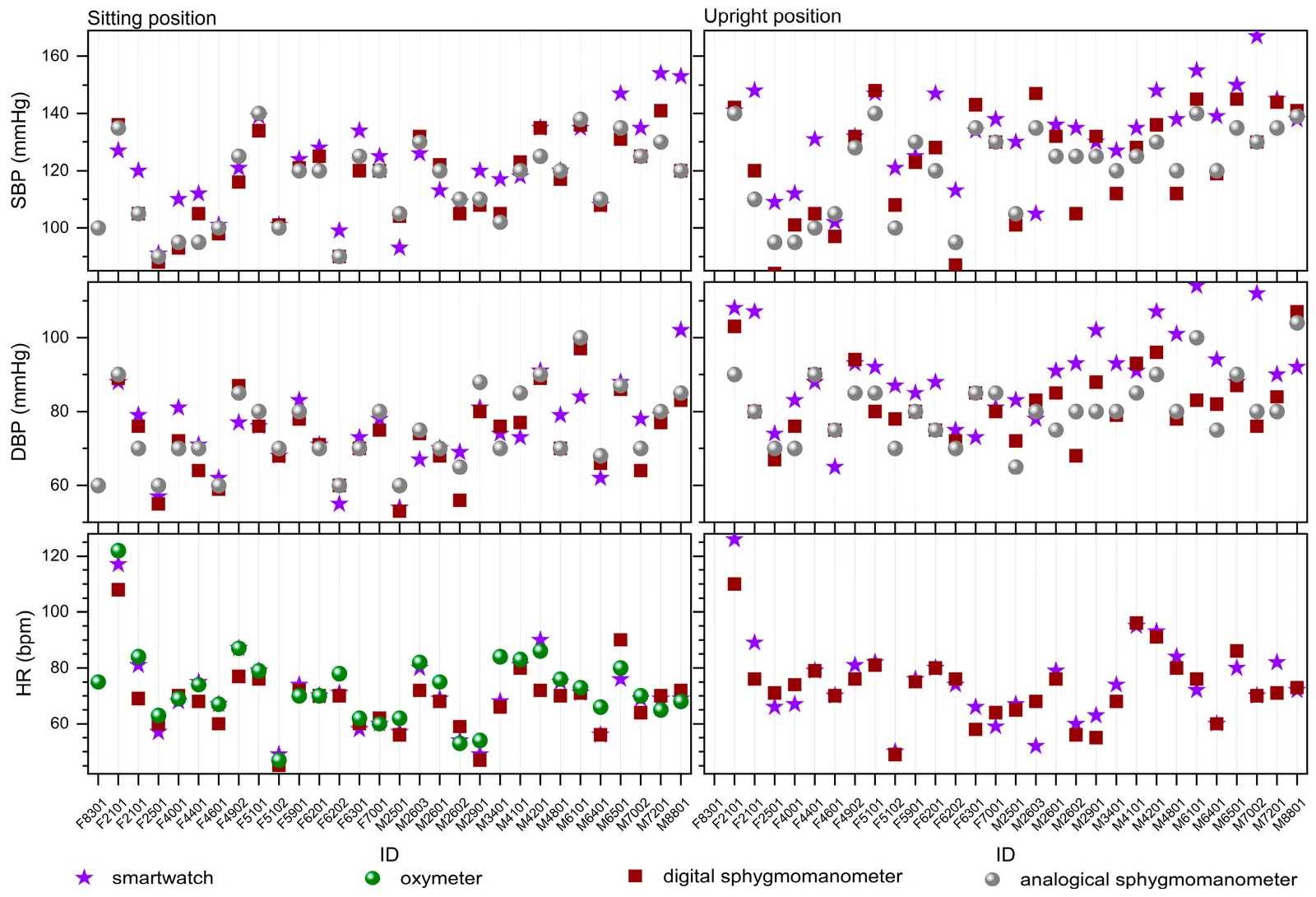
Measured values for all participants with the different instruments (smartwatch, oxymeter, digital and analogical sphygmomanometers). From top to bottom: systolic blood pressure (SBP), diastolic blood pressure (DBP), and heart rate (HR). Values while seated are shown on the left and in the upright position on the right.

**Figure 2:**
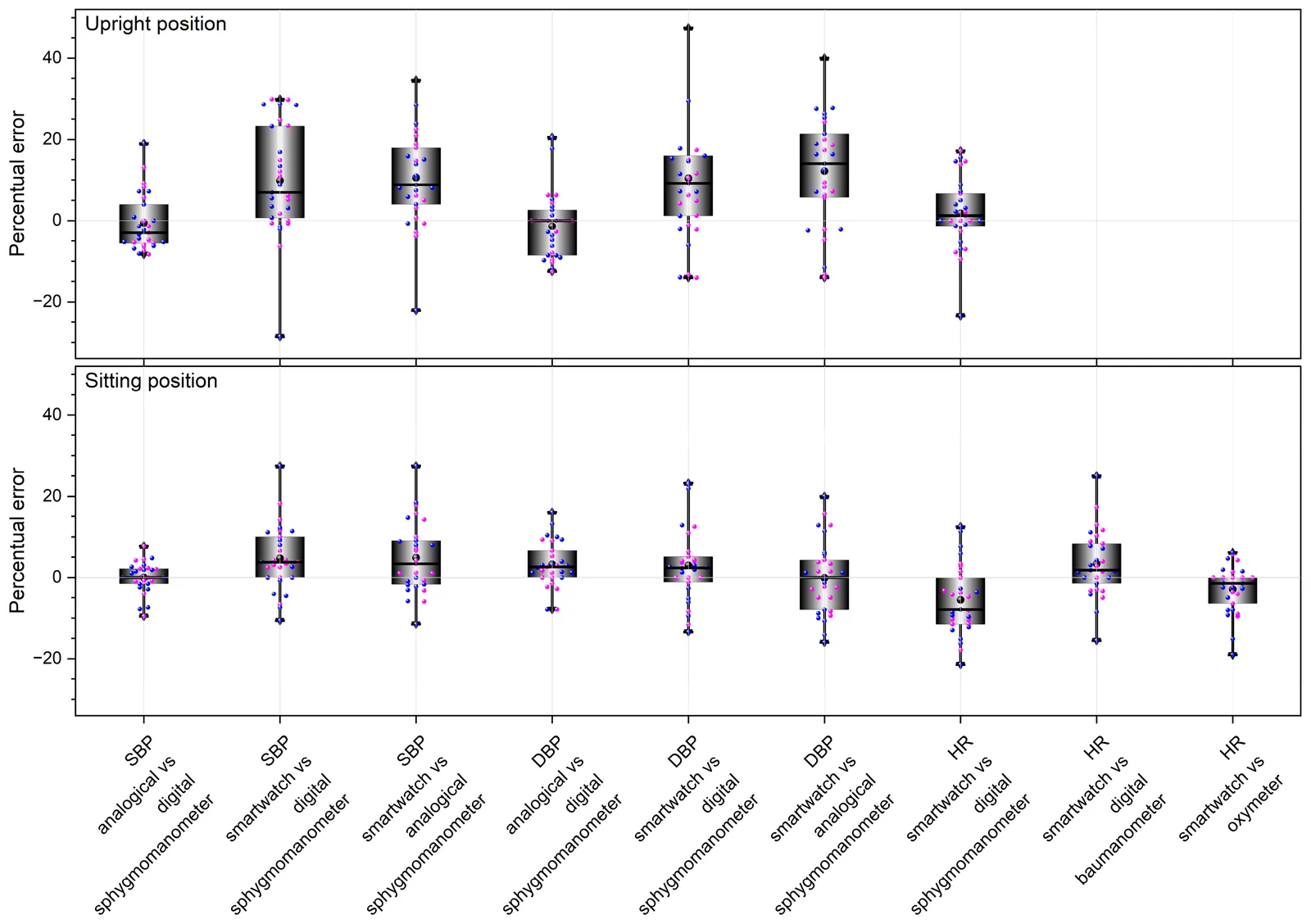
Percentage measurement error across instruments in the upright (top panel) and seated (bottom panel) positions. Percentage measurement error across instruments in the upright (top panel) and seated (bottom panel) positions. Blue points represent individual measurements from male participants, whereas pink points represent individual measurements from female participants. Gray box plots show the data distribution; the central line indicates the median (Q2), the box represents the interquartile range (Q1–Q3).

**Figure 3:**
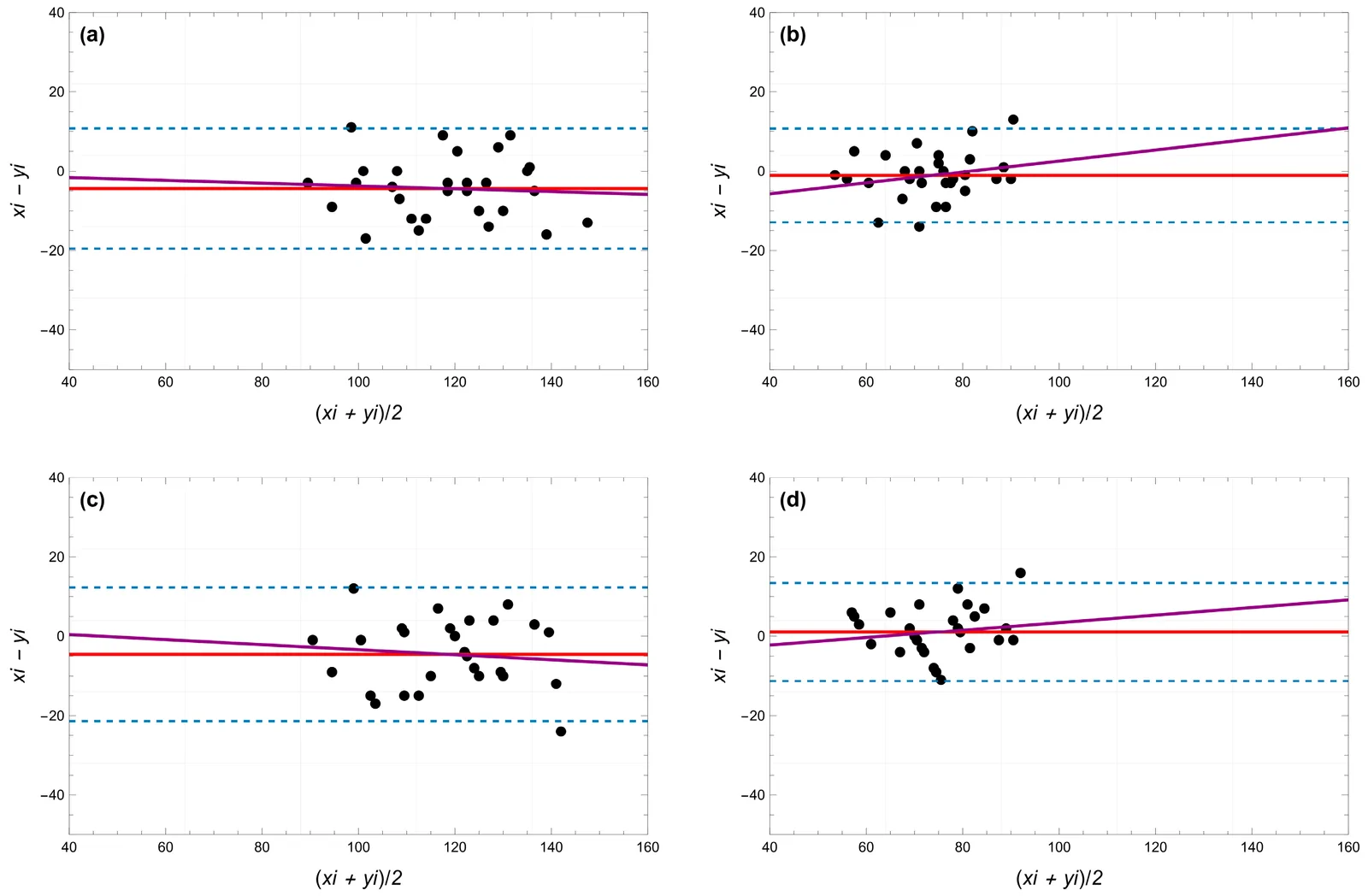
Bland–Altman plots of systolic blood pressure (SBP) comparing the digital sphygmomanometer (DS) and analog sphygmomanometer (AS) vs. HUAWEI WATCH D2 under seating position. Comparison between the digital sphygmomanometer vs. the smartwatch for systolic blood pressure (**a**) and diastolic blood pressure (**b**), with the comparison between analog sphygmomanometer vs. smartwatch for systolic pressure (**c**), and analog vs. smartwatch for diastolic pressure (**d**). In all panels, black points represent paired measurements, the red line indicates the bias, blue dashed lines the 95% limits of agreement, and the purple line the linear regression of the differences on the pair used to assess proportional bias.

**Figure 4:**
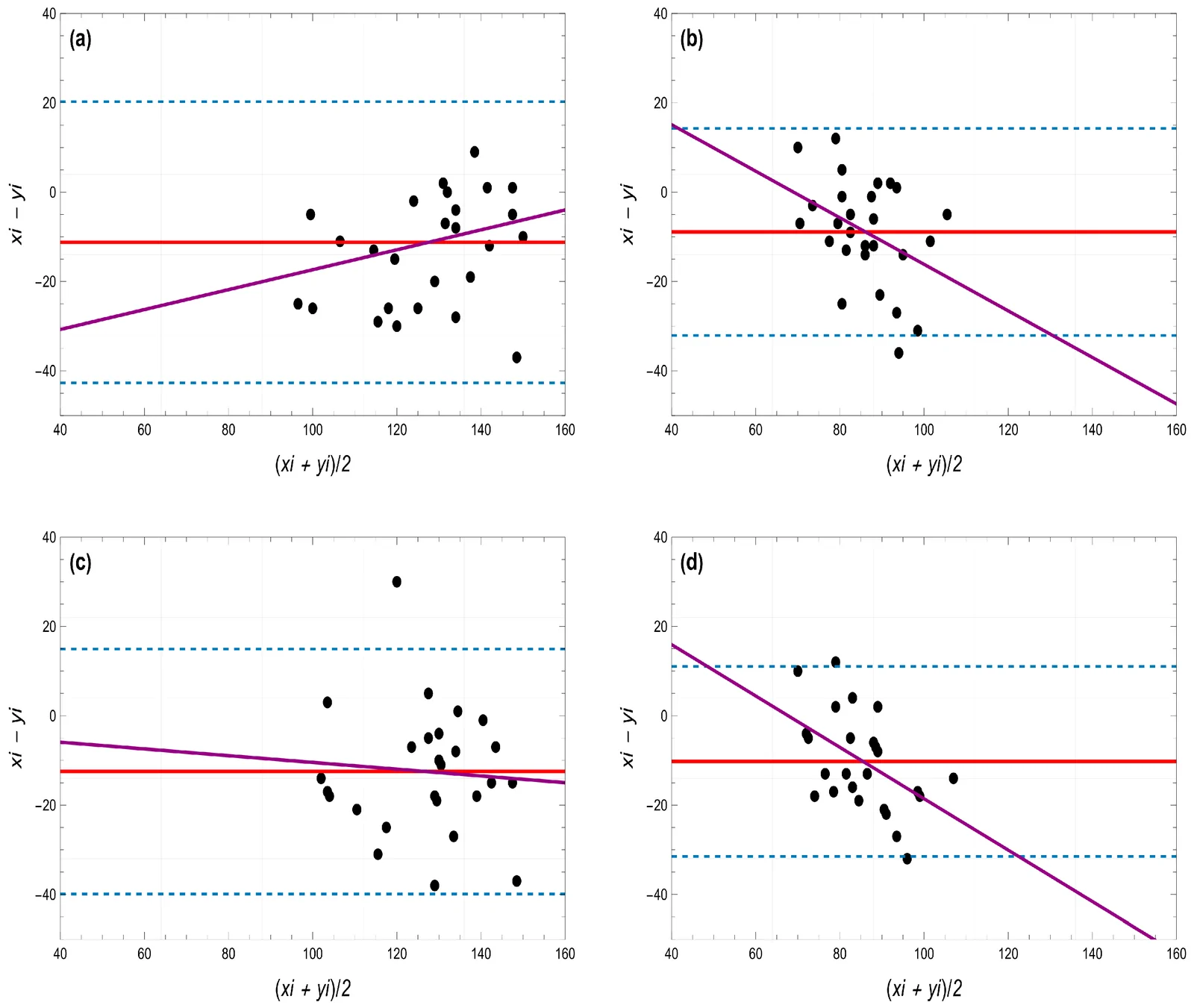
Bland–Altman plots of systolic blood pressure (SBP) comparing the digital sphygmomanometer (DS) and analog sphygmomanometer (AS) vs. HUAWEI WATCH D2 at upright position. Comparison between the digital sphygmomanometer vs. the smartwatch for systolic blood pressure (**a**) and diastolic blood pressure (**b**), with the comparison between analog sphygmomanometer vs. smartwatch for systolic pressure (**c**), and analog vs. smartwatch for diastolic pressure (**d**). In all panels, black points represent paired measurements, the red line indicates the bias, blue dashed lines the 95% limits of agreement, and the purple line the linear regression of the differences on the pair used to assess proportional bias.

**Table 1:** Percentual error of measurement values through different instruments.

				Median (Q1, Q3)	
				Sitting	Upright
Percentual error in SBP (analogical sphygmomanometer compared to digital sphygmomanometer)				0.0 (−1.5, 2.2)	−3.0 (−5.6, 4.0)
Percentual error in SBP (smartwatch compared to digital sphygmomanometer)				3.7 (0, 10)	6.9 (0.7, 23)
Percentual error in SBP (smartwatch compared to analogical sphygmomanometer)				3.3 (−1.7, 9.1)	8.8 (4.0, 18)
Percentual error in DBP (analogical sphygmomanometer compared to digital sphygmomanometer)				2.6 (0.0, 6.7)	8.8 (4.0, 18)
Percentual error in DBP (smartwatch compared to digital sphygmomanometer)				2.3 (−1.1, 5.1)	9.2 (1.1, 16)
Percentual error in DBP (smartwatch compared to analogical sphygmomanometer)				0.0 (−8.0, 4.3)	14 (5.7, 21)
Percentual error in HR (smartwatch compared to digital sphygmomanometer)				−8.0 (−11, 0.0)	1.2 (−1.4, 6.6)
Comparison	Q1 (Q2, Q3) Percentual error	Mean Error (Bias, mmHg)	SD of Error (mmHg)	95% Limits of Agreement (mmHg)	CCC (95% CI)
Sitting					
SW vs. DS (SBP)	3.7 (0, 10)	−4.4	8.16	−20 to +12	0.81 (0.70–0.87)
SW vs. DS (DBP)	2.3 (−1.1, 5.1)	−1.0	6.12	−13 to +11	0.80 (0.64–0.88)
SW vs. AS (SBP)	3.3 (−1.7, 9.1)	−4.5	8.93	−22 to +13	0.77 (0.66–0.85)
SW vs. AS (DBP)	0.0 (−8.0, 4.3)	+1.0	6.63	−12 to +14	0.77 (0.61–0.86)
Upright					
SW vs. DS (SBP)	6.9 (0.7, 23)	−10.0	15.31	−40 to +20	0.45 (0.12–0.65)
SW vs. DS (DBP)	9.2 (1.1, 16)	−8.0	11.48	−30 to +15	0.28 (0.04–0.54)
SW vs. AS (SBP)	8.8 (4.0, 18)	−12.0	14.03	−40 to +15	0.42 (0.17–0.59)
SW vs. AS (DBP)	14 (5.7, 21)	−10.0	10.71	−30 to +12	0.30 (0.09–0.48)

## Data Availability

The original contributions presented in this study are included in the article/Supplementary Material. Further inquiries can be directed to the corresponding author.

## Supplementary Materials

The following supporting information can be downloaded at https://www.mdpi.com/article/10.3390/healthcare14213566/s1. The site contains a database that includes punctual records of the cohort of 30 subjects in seated and upright positions. It includes oxygen saturation and heart rate (HR) registered by a digital pulse oximeter; systolic blood pressure (SBP), diastolic blood pressure (DBP), and HR recorded with a HUAWEI WATCH D2” smartwatch; SBP, DBP, and HR measured with a digital sphygmomanometer; SBP and DBP registered with an analog aneroid Hergom sphygmomanometer.

## Abbreviations

The following abbreviations are used in this manuscript:

BP	Blood Pressure
HR	Heart Rate
SBP	Systolic Blood Pressure
DBP	Diastolic Blood Pressure
SW	Smartwatch
DS	Digital Sphygmomanometer
AS	Analog Sphygmomanometer
CCC	Concordance Correlation Coefficient
LoA	Limits of Agreement
PE	Percentual Error
